# Cascade health service use in family members following genetic testing in children: a scoping literature review

**DOI:** 10.1038/s41431-021-00952-4

**Published:** 2021-08-26

**Authors:** Alexandra Cernat, Robin Z. Hayeems, Wendy J. Ungar

**Affiliations:** 1 Child Health Evaluative Sciences, Hospital for Sick Children Research Institute, Toronto, ON Canada; 2 Institute of Health Policy, Management and Evaluation, University of Toronto, Toronto, ON Canada

**Keywords:** Paediatrics, Genetic testing

## Abstract

Cascade genetic testing is the identification of individuals at risk for a hereditary condition by genetic testing in relatives of people known to possess particular genetic variants. Cascade testing has health system implications, however cascade costs and health effects are not considered in health technology assessments (HTAs) that focus on costs and health consequences in individual patients. Cascade health service use must be better understood to be incorporated in HTA of emerging genetic tests for children. The purpose of this review was to characterise published research related to patterns and costs of cascade health service use by relatives of children with any condition diagnosed through genetic testing. To this end, a scoping literature review was conducted. Citation databases were searched for English-language papers reporting uptake, costs, downstream health service use, or cost-effectiveness of cascade investigations of relatives of children who receive a genetic diagnosis. Included publications were critically appraised, and findings were synthesised. Twenty publications were included. Sixteen had a paediatric proband population; four had a combined paediatric and adult proband population. Uptake of cascade testing varied across diseases, from 37% for cystic fibrosis, 39% to 65% for hypertrophic cardiomyopathy, and 90% for rare monogenic conditions. Two studies evaluated costs. It was concluded that cascade testing in the child-to-parent direction has been reported in a variety of diseases, and that understanding the scope of cascade testing will aid in the design and conduct of HTA of emerging genetic technologies to better inform funding and policy decisions.

## Introduction

Cascade genetic testing is the systematic identification of individuals at risk for a hereditary condition by performing genetic testing in the family members of persons known to possess particular genetic variants (i.e., proband or index case) [1]. Three-generation pedigrees are often constructed as part of the baseline assessment of new index cases prior to genetic testing. This helps clinicians determine the most likely mode of inheritance and identify potentially at-risk family members. Following a positive genetic test in a proband, relatives are tested based on genetic proximity. They may also undergo cascade screening, involving imaging and other forms of testing, to determine whether any features of the condition under investigation are present. Probands and their family members typically receive both pre- and post-test counselling with a genetics expert (i.e., a genetic counsellor or molecular geneticist). The number and scheduling of genetic counselling sessions may vary from institution to institution.

Cascade health services are recommended for a variety of diseases. Early identification of at-risk individuals is important clinically, as it enables initiation or cessation of periodic screening/surveillance and may trigger lifestyle changes. Cascade testing can be performed as parent-to-child, with a parent identified as the index case and children undergoing carrier testing as indicated. When the proband is a child, cascade health service use occurs as child-to-sibling or child-to-parent. Even when parents do not present with signs or symptoms of disease, they undergo testing for segregation analysis [2] or to guide monitoring where symptoms could later develop.

In addition to prompting care for family members, cascade testing has implications for the health system because genetic testing can be costly and any consequential care is associated with additional health service use, such as physician referrals. Moreover, cascade service consumption may also result in improved quality or length of life in individuals beyond the proband since genetic testing provides an opportunity to improve surveillance and clinical management in patients’ families. Despite this, cascade effects are not traditionally considered in health technology assessment (HTA) or economic evaluations that form part of the evidence-base for funding and policy decision making. While guidelines for economic evaluation and HTA have begun to recognise the spillover effects of patients’ illness on the costs and health of caregivers [3], they do not address the costs and health effects from cascade services triggered by a patient’s phenotype or genotype. Rather, guidelines focus on the costs and health consequences of individual patients [4, 5]. A key step towards incorporating this aspect of genetic testing in HTA is understanding the patterns and costs of cascade health services in family members of index cases. While the uptake, costs, and downstream consequences of cascade genetic testing in adults have been explored [6–8], cascade effects of genetic testing in children have not yet been closely examined. As children are often the target of genetic testing and child-to-relative cascade screening is becoming more routine, it is important to examine this more closely. Distinguishing between adult and paediatric probands is important as uptake rates and patterns of cascade health service use may be different. Moreover, in HTA, ethical considerations and modelling of long-term costs and consequences of cascade services must be distinct for children and adults. Future research must consider different scenarios for cascade services related to the needs of particular patient groups as well as population-based targeted screening for rare diseases. The aim of this scoping review was to characterise the patterns and costs of cascade health service use by relatives of children with any condition diagnosed through genetic testing.

## Materials and methods

### Study design

This scoping review was guided by the following research questions: What is the rate and pattern of uptake of cascade testing or screening of family members of children who receive a genetic diagnosis? What are the costs and downstream health services associated with this type of cascade testing and screening? While the purpose of a systematic review is to arrive at a “critically appraised and synthesised answer to a particular question” [9], scoping reviews “aim to map rapidly the key concepts underpinning a research area … especially where an area … has not been reviewed comprehensively before” [10]. Given that the care and cost consequences of cascade testing and screening stemming from a paediatric proband have not been widely explored, a scoping review was conducted.

### Search strategy and eligibility

Ovid Medline and Embase were searched for studies published from January 1, 2000 to January 8, 2020 using keywords, MeSH terms, and Emtree subject headings (Supplementary Tables S1 and S2). The electronic search was supplemented with a manual search of reference lists of eligible papers.

Eligible publications were in English and reported quantitative findings regarding the uptake, costs, downstream consequences or health service use, or cost-effectiveness of cascade testing or screening of family members of children receiving a genetic diagnosis, even if assessing cascade testing was not the primary study aim. Studies reporting the index case as paediatric, or a combination of paediatric and adult were eligible. Ineligible studies included papers in languages other than English, and animal, in vitro, or purely qualitative research. Studies in which paediatric index cases were clinically rather than genetically diagnosed, or the method of diagnosis was unspecified were excluded. Titles and abstracts were reviewed by one reviewer and full-text articles were obtained for eligible studies. A Preferred Reporting Items for Systematic Reviews and Meta-Analyses (PRISMA) flow diagram was constructed [11].

### Data extraction and analysis

For each study, bibliographic information, purpose, design, methodology, and findings were extracted by one researcher. The literature was categorised according to disease type and findings were summarised descriptively.

### Critical appraisal

Included papers were critically appraised using the Scottish Intercollegiate Guidelines Network (SIGN) critical appraisal checklist appropriate to the study design [12]. Details about this process are provided in Supplementary File A.

## Results

**Fig. 1:**
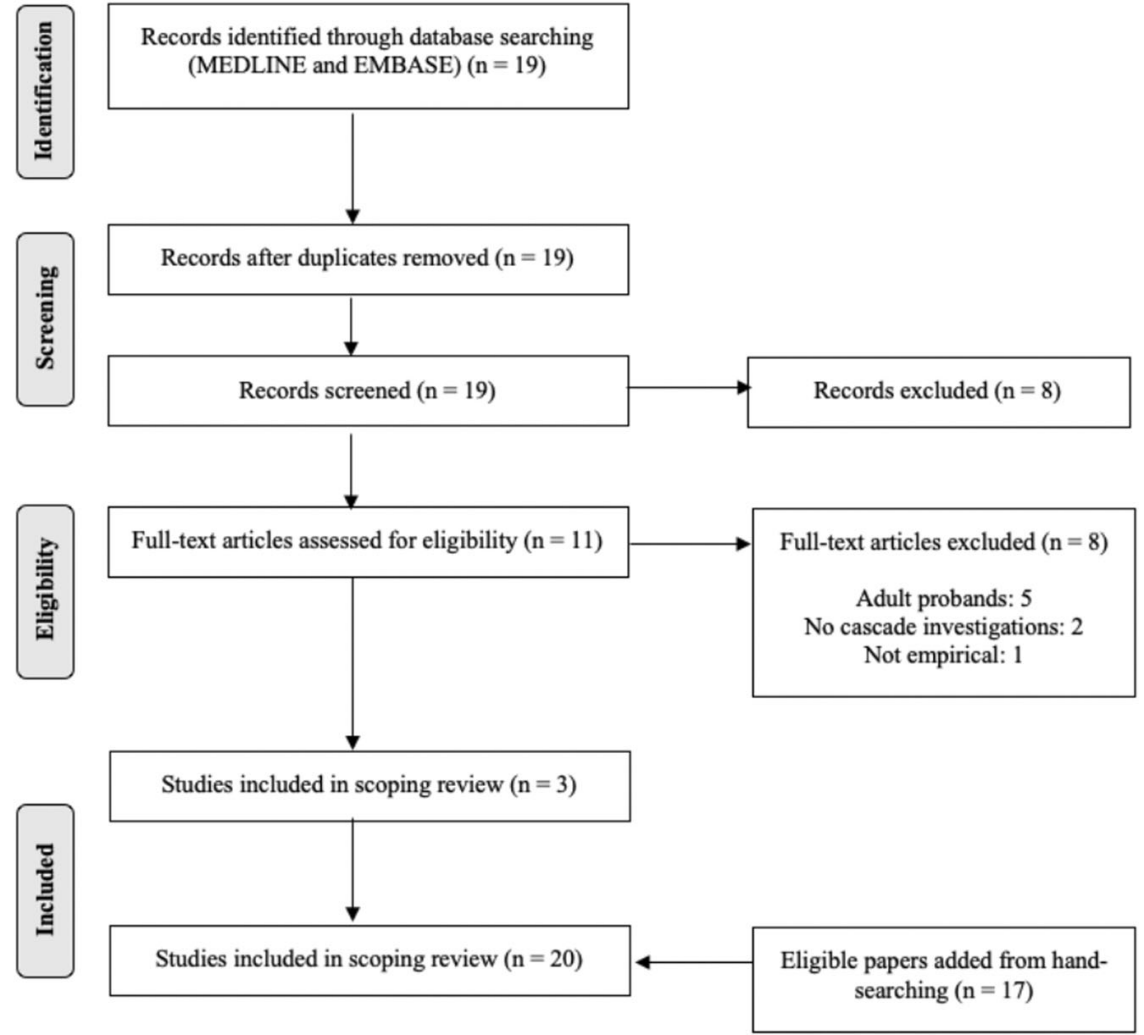
PRISMA flow diagram. Nineteen records were identified through an electronic search of Medline and Embase, of which 11 proceeded to full-text review. An additional 17 articles were retrieved through hand-searching. A total of 20 studies were included in the review.

The search yielded 19 publications of which 11 proceeded to full-text review and 17 articles were retrieved through hand-searching. In total, 20 studies were included (Fig. 1). The studies were grouped into cardiovascular, haematologic, and other monogenic conditions, and are summarised in Tables 1–3. Most studies focused on uptake of testing among probands’ relatives [13–17] and only two studies assessed costs [18, 19]. The included studies were conducted in Australia [14, 17, 19], China [15], India [13], the United Kingdom [20], and the United States [16, 21–23].

**Table 1:** Included studies conducted in cardiovascular conditions.

Author [ref.]	Study location	Disease state	Focus of study	Proband population	Included relatives	Main findings/conclusions
Knight et al. [16]	United States	LQTS and HCM	• Uptake and yield of genetic testing in children with LQTS or HCM • Uptake and yield of cascade genetic testing	• 168 children with LQTS, mean age ± standard deviation (SD): 8.4 ± 5.7 years • 147 children with HCM, mean age ± SD: 9.4 ± 6.1 years	• 553 relatives	Probands: • 92% with LQTS underwent genetic testing; 81% positive • 65% with HCM underwent genetic testing; 60% positive Relatives: • 46% clinical screening only; 38% clinical screening and genetic testing; 17% genetic testing only → 1.6 cascade interventions per relative • 40% of all relatives positive • Larger proportion positive among LQTS families than HCM families (42% vs. 37%) • Higher yield with combined cascade screening and genetic testing than cascade screening or genetic testing alone
Truong et al. [26]	Vietnam	FH	• Outcomes of cascade genetic testing	• 2 children, aged 4 and 8 years • 3 adults, aged 28, 33, and 42 years	• 107 first- and second-degree relatives from 4 families	Relatives: • 83% underwent cascade genetic testing; 52% positive
Wu et al. [15]	China	FH	• Yield of cascade genetic testing and screening	• 47 consecutive paediatric patients, mean age ± SD: 9.1 ± 4.8 years	• 70 parents • 10 siblings • 46 second-degree relatives	Relatives: • 100% first-degree positive and 89% second-degree positive → mean yield of cascade genetic testing: 2.8 new FH cases per proband
Wald et al. [20]	United Kingdom	FH	• Feasibility of child-to-parent FH screening in primary care settings	• 10,095 children tested, median age: 12.7 years; 37 children positive for FH variant; 32 participated	• 64 parents	Parents: • 63% positive • For every 1,000 children screened, 8 individuals (4 children and 4 parents) had positive cascade test or screen
Alfares et al. [18]	United States	HCM	• Results of genetic testing for non-syndromic HCM in probands and family members	• 2,912 paediatric and adult probands referred for HCM genetic testing, no age information available	• 1,209 asymptomatic relatives	Probands: • 32% positive (28% paediatric) • 15% inconclusive Asymptomatic family members of mutation-positive probands: • 57% negative → no longer needed cardiac evaluations → health systems savings of ~US $1,000 per relative
Miller et al. [24]	United States	HCM and DCM	• Uptake of cardiac screening and genetic testing among at-risk relatives • Factors influencing uptake of cascade genetic testing	• 57 paediatric and adult probands (46 HCM and 11 DCM), mean age (range): 15 (0.02–64) years	• 302 first- and second-degree relatives	Probands: • 40/57 positive Relatives of mutation-positive probands: • 39% underwent cascade genetic testing • 59% underwent cascade screening • Uptake of cascade services greater in first-degree than second-degree relatives • No statistically significant association between proband’s age at diagnosis, family history of SCD, and number of living affected relatives, with uptake of cascade genetic testing • No statistically significant association between proband’s genetic testing results and uptake of cascade clinical screening
Leren et al. [25]	Norway	FH	• Outcome of cascade genetic testing	• 188 paediatric and adult index patients	• 851 relatives, “primarily” first-degree	Relatives: • 47.9% positive • 78/146 affected relatives used test results to change medications

*DCM* dilated cardiomyopathy, *FH* familial hypercholesterolaemia, *HCM* hypertrophic cardiomyopathy, *LQTS* long QT syndrome.

**Table 2:** Included studies conducted in haematologic conditions.

Author [ref.]	Study location	Disease state	Focus of study	Proband population	Included relatives	Main findings/conclusions
Tairaku et al. [29]	Japan	• Severe congenital protein C deficiency	• Outcome of prenatal diagnosis in sibling of affected child	• 1 child, aged 3 years	1 foetus in utero	Foetus: • Heterozygous carrier; would not experience symptoms
Gorakshakar and Colah [13]	India	• β-thalassaemia	• Uptake and results of cascade screening	• Paediatric, number of affected children not specified 490 children from “high risk” communities, ages not reported	• 691 relatives from 44 families, including 25 siblings of index cases	Children from “high risk” communities: • 96/490 (20%) heterozygotes Relatives: • Among siblings of index cases, 10/25 (40%) heterozygotes
Baig et al. [30]	Pakistan	• β-thalassaemia	• Cascade genetic testing results	• 1 child, age not reported	• 27 relatives	Relatives: • 44.4% carriers
Cadet et al. [28]	France	• HH	• Yield of cascade testing and screening of at-risk adults identified through neonatal screening of infants	• Neonatal (number not specified)	• 11 families of *C282Y*^a^ homozygous infants • 10 families of heterozygous infants • Number/type of relatives not described	Families of homozygous infants: • 5 relatives from 4 families homozygous Families of heterozygous infants: • 5 relatives from 2 families homozygous • 6/10 homozygous relatives began treatment; 4/10 homozygous relatives under surveillance

*HH* hereditary hemochromatosis.

^a^HH-conferring variant.

**Table 3:** Included studies conducted in other monogenic conditions.

Author [ref.]	Study location	Disease state	Focus of study	Proband population	Included relatives	Main findings/conclusions
Stark et al. [19]	Australia	Rare monogenic disorders	• Clinical and cost impacts of genomic sequencing in infants with suspected monogenic disorders	• Paediatric (number not specified), mean age (range): 8 months (1 week–34 months)	• 88 first-degree relatives	Relatives: • 90% underwent genetic testing (total cost: AU $28,000) • 2 first-degree relatives changed medical management based on genetic test results (yearly costs: AU $146 and AU $329) • 16 couples accessed reproductive genetic services (total cost: AU $56,904.37)
Famula et al. [21]	United States	Fragile X syndrome	• Identification of affected child through newborn screening • Outcome of cascade genetic testing	• 1 child, aged 3 months	• 3 family members (mother and 2 siblings)	Relatives: • all 3 relatives found to have full *FMR1* (fragile X-associated) variant
McClaren et al. [17]	Australia	CF	• Uptake of relative carrier testing and factors influencing uptake	• 30 children, ages not reported	• 225 relatives	Relatives: • 37% underwent carrier testing
Sorensen et al. [23]	United States	Fragile X syndrome	• Description of pilot project: newborn screening followed by cascade testing • Detailed description of 3 newborns identified as having Fragile X syndrome premutation	• 3,024 newborns screened; 14 positive • 3 newborns described in detail, aged 5 months, 5 months, and 6 months	• 44 relatives of variant-positive probands	Relatives: • 27/44 (61%) positive
Moriwaki et al. [32]	Japan	XP-A	• Experience of 1 centre with prenatal diagnosis of XP-A	• 12 children from 9 families, mean age (range): 3.83 (1–11) years	• 10 fetuses in utero	Fetuses: • 2/10 XP confirmed • 6/10 XP carriers • 2/10 unaffected
Sorensen et al. [22]	United States	Fragile X syndrome	• Description of fragile X syndrome sibship • Outcome of cascade genetic testing	• Brother and sister pair; brother was true proband, aged 9.75 years	First- and second-degree relatives (number not specified)	Relatives: • Parents both carriers • Third sibling unaffected • Maternal grandmothers obligate carriers
McClaren et al. [14]	Australia	CF	• Uptake of cascade genetic testing by non-parent adult relatives	• 30 children, ages not reported	• 59 parents • 716 non-parent first- and second-degree relatives	Parents: • 64.4% underwent genetic testing Non-parent relatives: • 11.5% underwent genetic testing • 2.7 relatives tested per child • Female relatives 1.61 times more likely than males to undergo cascade testing
Smith et al. [31]	Australia	SMA	• Carrier frequency of SMA in Australia	• Paediatric (number not specified), ages not reported	• 117 parents of affected children • 158 individuals with family history • 146 individuals without family history	• SMA carrier frequency ~1/49
Rudolph et al. [33]	Germany	X-linked ocular albinism	• Outcomes of genetic testing and clinical screening	• 1 male, aged 8 months	• 22 relatives	Family members: • 6 male relatives affected • 6 other relatives identified as obligate carriers

*CF* cystic fibrosis, *SMA* spinal muscular atrophy, *XP-A* xeroderma pigmentosum complementation group A.

### Cardiovascular conditions

Seven studies were conducted in cardiovascular conditions, including hypertrophic cardiomyopathy (CMP) (HCM) [16, 18, 24], dilated CMP (DCM) [24], long QT syndrome (LQTS) [16] and FH [15, 20, 25, 26] (Table 1). A systematic family screening programme for FH was established in Norway in the early 2000s [25]. After three years, 851 relatives of 188 probands had undergone genetic testing, and 407 (47.9%) were affected. Relatives of both paediatric and adult probands were included, but the number of index patients in either age group was not specified, contributing to uncertainty in the findings. This paper was included because cascade genetic testing was triggered in the families of paediatric index FH patients. Little data on the probands were presented, and reasons for initially testing children were not provided. Biochemical testing was performed, with total serum cholesterol of probands and relatives measured before lipid-lowering drugs were started. For included relatives (both affected and unaffected), the mean total serum cholesterol, HDL-cholesterol triglyceride, and LDL-cholesterol levels were also measured and reported. A follow-up survey in 146 affected relatives found that approximately half had made changes to their medications based on their genetic results [25]. Some data were available for these 146 relatives. Lipid values were measured at the time of genetic testing, as well as six months after testing, and there was a statistically significant decrease in their total serum cholesterol and LDL-cholesterol levels, as well as a statistically significant increase in their HDL-cholesterol levels. No information was provided for relatives who made changes to their drug therapy subsequent to genetic testing.

Miller et al. [24] investigated screening and genetic testing in families of paediatric and adult patients with HCM or DCM in the US. The cohort consisted of 57 probands and 302 relatives recommended to undergo cardiac screening, genetic testing, or both. Eighty-one percent and 19% of probands had HCM and DCM, respectively. Most (70%) probands who underwent genetic testing had a pathogenic (i.e., definitively causative [27]) variant. Genetic testing for a familial variant was indicated for 213 relatives (140 first-degree and 73 second-degree) of variant-positive probands. Seventy-two first-degree (51%) and 12 second-degree (16%) relatives accepted the offer of testing. A greater proportion of first-degree compared to second-degree relatives underwent both cardiac screening (83% vs. 30%) and genetic testing (51% vs. 16%).

Alfares et al. [18] performed genetic testing in 2,912 unrelated paediatric and adult probands, and familial variant testing in 1,209 of their asymptomatic relatives to identify HCM-associated variants and to assess the costs associated with cascade testing. Resource use was not measured empirically. A pathogenic or likely pathogenic variant was identified in 917 of 2,192 probands (32%) and testing was inconclusive in 444 individuals (15%). Twenty-eight percent of positive genetic tests were in probands 16 years old or younger. Among the asymptomatic relatives of variant-positive probands, 691 received a negative result and no longer required cardiac surveillance, equivalent to savings of about US $1,000 per relative. The number of tested relatives related to a paediatric vs. adult proband was not reported.

Wald et al. [20] screened over 10,000 British children aged 1–2 years for FH. Children had positive screens if they tested positive for a genetic variant or had elevated cholesterol three months after initial testing [20]. The parents of children with positive screens were tested for FH-associated variants. A positive result was defined as having the same variant as their child or having a higher cholesterol level than the other parent. Of the 10,095 included children, 37 (0.37%) were genetically diagnosed with FH. Both parents of 32 of these children underwent clinical screening and genetic testing. Most parents who tested positive but were not yet receiving statins began treatment based on the results. Early identification and treatment of affected relatives was highlighted as one of the benefits of identifying children with FH at an early age.

In an FH study in Beijing [15], the first- and second-degree relatives of 47 children with genetically diagnosed FH underwent cascade genetic testing. FH was diagnosed in 12 of the tested relatives (2.8 new cases per proband), but the proportion of cases identified in parents compared with second-degree relatives was not reported.

In a case series describing outcomes of cascade genetic testing and clinical screening for FH in Vietnam [26], five index patients (two children and three adults) and 107 relatives underwent cascade investigations. Of these family members, 89 agreed to genetic testing. An FH-associated variant was found in 47 individuals.

Finally, Knight et al. [16] examined the uptake and yield of cascade genetic testing in the family members of children with LQTS and HCM across six paediatric centres in the US. A total of 315 index patients from 315 families were identified, and genetic testing was performed in 250 (79%). The index patient was the first family member seen at the participating centre. Uptake was higher among LQTS than HCM patients (92% vs. 65%). Of tested index patients, 81% with LQTS and 60% with HCM received a positive result. Of the 315 families included, 234 (74%) agreed to cascade genetic testing and/or screening, with a total of 553 relatives (2.4 per family) undergoing cascade investigations. Participation in cascade testing or screening was highest among families with a variant-positive index patient (90%), but 67% of families in which the index patient received a negative or inconclusive genetic test result agreed to screening. It was not specified whether this cascade screening involved clinical screening only, genetic testing or both. Uptake of cascade investigations among families in which the index case did not undergo genetic testing or had an unknown genetic testing status was 43%. Overall, a mean of 1.6 cascade investigations were performed per relative screened, with 17% or relatives undergoing cascade genetic testing only, 46% undergoing cascade clinical screening only and 38% undergoing both. Although all relatives of variant-positive index patients were eligible for cascade genetic testing, 26% chose clinical screening only. In total, 221/553 relatives (40%) were affected (0.94 relatives per family). Of the three screening strategies (genetic testing only, screening only, and both), the combined strategy had the highest yield (58%) compared with genetic testing only (34%) and screening only (19%).

### Haematologic conditions

Four studies addressed haematologic conditions such as hereditary hemochromatosis (HH) [28], severe congenital protein C deficiency [29], and β-thalassaemia [13, 30] (Table 2). Cadet et al. [28] explored the effectiveness of “reverse cascade screening” to identify adults at risk for HH. The authors screened 7,038 newborns for *C282Y* and *H63D* (HH-conferring) variants. Nineteen infants from 18 families were *C282Y* homozygotes and 657 infants were *C282Y* heterozygotes. Eleven (61%) of the families of homozygous children underwent cascade genetic testing and ten (1.6%) families of heterozygous children requested cascade investigations. Ten relatives were *C282Y* homozygotes. Cadet et al. concluded that neonatal screening is more effective than untargeted screening of adults for detection of HH.

Gorakshakar and Colah [13] contacted relatives of children with β-thalassaemia across Mumbai to offer genetic testing. Six hundred and ninety-one family members underwent testing and 151 (22%) were identified as carriers. Targeted cascade genetic testing was found to be five to six times more effective than untargeted community-based genetic testing to identify carriers. Finally, there were two case reports of individual families in which a child was genetically diagnosed with β-thalassaemia [30] or severe congenital protein C deficiency [29], and relatives underwent genetic testing.

### Other monogenic conditions

Nine studies examined other monogenic conditions, such as Fragile X syndrome [21–23], cystic fibrosis (CF) [14, 17], spinal muscular atrophy (SMA) [31], xeroderma pigmentosum complementation group A (XP-A) [32], and X-linked ocular albinism [33] (Table 3). Smith et al. [31] conducted cascade testing in 117 parents of children with SMA in Australia. In addition to parents, 158 and 146 unaffected individuals with and without a family history of SMA, respectively, were included. Of the tested parents, 94% were carriers. Forty-seven percent of those with a family history and 2% of those without a family history were carriers.

In Australia, uptake of cascade genetic testing after diagnosis of a newborn with CF was 16.3% for all relatives including parents and 11.8% in non-parent relatives [14]. Larger families (20 or more members) had lower uptake (15.4%) than smaller families (19.6%), and on average, three non-parent relatives had carrier testing per child. Female relatives were 1.6 times more likely than males, and first-degree relatives were five times more likely than second-degree relatives, to undergo testing. Uptake of cascade genetic testing differed among family members: parents, 64.4%; grandparents, 23.4%; aunts or uncles, 38.9%; first cousins, 15.4%; and half-siblings, 50%. Most non-parent relatives (88.5%) offered carrier testing declined. A follow-up study [17] with 225 relatives of these children revealed the most common reasons non-parent relatives did not pursue cascade testing included already having their children, not thinking about cascade testing, not having an immediate need to undergo testing or not being offered testing.

In a Japanese study of prenatal diagnosis for XP-A in ten families [32], two foetuses were affected, six were carriers, and two were unaffected.

Sorensen et al. [23] reported on a pilot project for newborn screening and cascade testing for *FMR1* (Fragile X syndrome-associated) variants in the US. As of 2013, 3,042 newborns were screened, and 44 family members of genotype-positive newborns underwent testing. In all, 14 newborns and 27 relatives from ten families were variant-positive. Sorensen et al. also presented a case series of three newborns identified as having premutations in *FMR1* where carrier testing was performed in relatives including parents, aunts and uncles, grandparents, and great-grandparents [23].

Stark et al. [19] investigated the longer-term clinical and health economic impacts of exome sequencing for rare diseases in 80 infants with suspected monogenic disorders. They investigated the uptake and cost of cascade testing among first-degree relatives, the cost and impact of any changes in the medical management of these relatives based on their cascade testing results, and the use of reproductive genetic services among first-degree relatives. Of 88 eligible relatives, 79 (90%) accepted testing with a total cost of AU $28,000. Additionally, two asymptomatic first-degree relatives experienced a change in medical management, resulting in additional costs of AU $146 and AU $329. Stark et al. also assessed the use of reproductive genetic services by 16 couples (14 with diagnosed children and two with undiagnosed children). Of the couples with diagnosed children, three sought pre-implantation genetic diagnosis (PGD), and two of them proceeded with it. The 11 other couples with diagnosed children sought prenatal diagnostic services; four of these accessed them. The cost of PGD was AU $29,804, and prenatal diagnostic services AU $27,100.

Case reports of individual families in which a child received a genetic diagnosis of Fragile X syndrome [21, 22] or the Nettleship-Falls type of X-linked ocular albinism [33] have also been conducted and genetic testing in the child’s relatives was described.

### Critical appraisal of included literature

Studies were categorised as high quality, acceptable or low quality (Supplementary Tables S3 and S4). The majority of appraised studies were acceptable, with one [13] considered low quality. The findings of the critical appraisal are described in full in Supplementary File A.

## Discussion

This scoping review characterised the research to-date related to the pattern and costs of cascade health service use by the families of children with any condition diagnosed using genetic testing. The 20 included studies were conducted in a variety of diseases, including CMP [16, 18, 24], FH [15, 20, 26], and HH [28]. One study [19] had a broader focus and was concerned with infants potentially affected by any rare monogenic disorder. Determining a genetic basis of disease can help guide clinical management or establish a prognosis of the patient and can inform surveillance of their families. For example, paediatric CMP patients might have extremely poor prognoses depending on the genetic variant(s) they possess [34, 35]. A molecular diagnosis could help initiate treatment sooner. One study was conducted in HH [28]. Since HH is typically adult-onset [36], initiating carrier testing in asymptomatic newborns to identify at-risk adults may not align with practice guidelines in all jurisdictions.

sDisease mode of inheritance, penetrance, and expressivity are important factors to consider with respect to cascade genetic testing or clinical screening. Studies included in this review focused on diseases typically inherited in an autosomal dominant manner such as LQTS [16], HCM [16, 18, 24], DCM [24], and FH [15, 20, 25, 26], or in an autosomal recessive fashion like β-thalassaemia [13, 30], CF [14, 17], and XP-A [32]. Several studies examined diseases with an X-linked recessive mode of inheritance such as X-linked ocular albinism [33] or an X-linked dominant inheritance pattern such as Fragile X [21–23]. Finally, some of the diseases under study, specifically severe protein C deficiency [29], HH [28], and SMA [31], follow multiple inheritance patterns, depending on the specific genetic variant present. In the case of autosomal dominant conditions with high penetrance, it may be relatively simple to assess relative risk status with pedigree construction. In contrast, for autosomal recessive conditions, even those with high penetrance such as CF, cascade genetic testing or screening is important because it may be difficult to identify heterozygous carriers in a family through pedigree construction alone. Regardless of inheritance pattern, identifying at-risk family members is complicated if diseases display incomplete penetrance or variable expressivity (e.g., LQTS, CMP, FH, β-thalassaemia, Fragile X, severe protein C deficiency, and HH) as genotype and phenotype do not always correlate [37]. Asymptomatic individuals may still be at risk and could benefit from monitoring. Information from cascade testing and screening may have clinical utility such as initiation of primary prevention (for instance, early initiation of statin treatment for FH), but it may also have personal utility since information about carrier status may have implications for family planning, especially for relatives at a reproductive age.

Studies mainly reported uptake or yield of cascade genetic testing in probands’ relatives. Uptake of cascade testing in relatives of paediatric HCM patients ranged from 39% [24] to 65% [16]. In contrast, uptake of cascade testing by relatives of children with CF was 37% [17], while uptake among relatives of infants suspected to have a rare monogenic condition was 90% [19]. Uptake may be partly influenced by a condition’s penetrance, expressivity, and inheritance pattern. The clearer the relationship is between genotype and phenotype, the easier it may be for an individual to infer their carrier status based on the genotype of a relative, reducing the need for testing [17]. Disease treatability may also play a role in uptake as the absence of treatment options may be a barrier to testing in risk-averse individuals. However, it appears that the potential for receiving non-medically actionable results does not deter individuals from undergoing genetic testing. A study in individuals receiving genome sequencing results found that even though some participants were concerned about the psychological impact of receiving results for untreatable diseases, the majority would still want to receive them because “the benefits of knowing outweigh the risks of being fearful” [38].

In one study [16], 67% of families with a variant-negative or variant-inconclusive LQTS or HCM proband agreed to cascade screening. “Cascade screening” was used to describe both genetic and non-genetic screening, and Knight et al. [16] did not specify what proportion of families of variant-negative or -inconclusive index cases underwent each type of screening. While clinical practice guidelines for HCM suggest relatives of all index cases, regardless of proband genotype status, undergo cascade screening, they stipulate genetic testing should only be performed in the relatives of variant-positive index patients [39, 40]. Cascade screening of all relatives may be important because individuals can be genotype-negative but phenotype-positive [16]. There are also reasons why cascade genetic testing in relatives of genotype-negative or genotype-inconclusive probands may be warranted. For instance, inconclusive results may indicate a variant of unknown significance (VUS). Assessing whether relatives possess the same VUS could elucidate disease aetiology and may help determine the pathogenicity of the variant.

Only two included publications addressed costs [18, 19]. Of them only one [19] was an economic evaluation. Alfares et al. [18] estimated the cessation of cardiac surveillance in genotype-negative relatives of HCM probands translated into savings of US $1,000 per relative. Stark et al. [19] found that the total cost of cascade genetic testing in the parents of infants with suspected monogenic conditions was AU $28,000. Studies with adult CMP patients as probands have found the lifetime cost per patient for cascade genetic testing is between €19,459 and €21,803 (2007 currency) [41]. Multiple studies [6, 41, 42] have found that cascade genetic testing is cost-effective compared with cascade clinical screening alone for CMP. Similarly, studies [43, 44] have explored the cost-effectiveness of cascade genetic testing in family members of FH index cases compared with no cascade testing. Ademi et al. [43] found that, over a lifetime, cascade testing was cost-saving by approximately AU $1,100 per person. Ademi et al. [44] found that the cost of cascade screening for FH is approximately AU $1,600 per person.

Though economic evaluation guidelines have begun to recognise various forms of spillover effects, cascade effects of genetic testing are not currently incorporated in HTA [3–5]. However, the included studies indicated that the health system consequences of cascade health service use in the families of children with genetic conditions may be substantial. Economic evaluations of emerging testing technologies may therefore underestimate the costs and health benefits attributable to the implementation of genetic or genomic technologies in clinical care. The findings of this review thus underscore the importance of including health service use and costs triggered by the genetic testing of an index patient in HTA. Ongoing research is investigating the costs and use of cascade services in family members of children with CMPs, FH, familial adenomatous polyposis, and unexplained developmental delay, as well as the data and methodological challenges for incorporating cascade effects in HTA and economic evaluation. Future research must develop and validate formal methods to enable inclusion of cascade costs and health effects. In addition, future research should purposefully collect cascade testing and screening data to generate evidence for use in HTA not limited to modelling.

A strength of this review was that inclusion was not limited by disease. Additionally, a variety of study types were included, enabling a better understanding of the state of research on cascade testing prompted by a genetic diagnosis in a child. Qualitative works were excluded as they likely would not have provided specific data on uptake, yield, or costs of cascade service use. This is a limitation: understanding families’ perspectives would provide important context related to the uptake of these services. Moreover, in some studies with a combined paediatric and adult proband population, the number of index cases from each age group whose relatives underwent cascade investigations was not provided [18, 25]. As a result, it was not always possible to report the full implications of cascade testing or screening triggered by genetic testing of children alone. However, such studies were a minority, so they are not expected to have a large impact on the overall results of this review. Five studies [13, 16, 19, 24, 30] were unclear regarding the extent of genetic testing in probands or cascade testing and screening in relatives. However, the effects on the results of this review are expected to be minor as one of the inclusion criteria was that cascade health service use had not been triggered by clinical screening or index case phenotype alone.

An additional challenge was developing a comprehensive search strategy and some eligible papers may have been missed. Of included studies, only three were identified through an electronic search of Medline or Embase, and the remaining 17 were found manually. When designing the search, emphasis was placed on capturing the idea of a paediatric proband. However, it is possible that the focus on a child as the index patient may have compromised the identification of papers with a combined paediatric and adult proband population. In addition, there is no MeSH term or Emtree subject heading for cascade genetic testing, so there is no index for articles specifically about this topic. There is wide variation in the keywords authors use to describe cascade testing, for instance, some call it cascade testing or cascade screening [13, 15, 16, 23, 28, 31]; others refer to it as carrier screening [14, 17]; others still describe it as family screening [45]. These terms were included as terms in the search strategy, but they do not appear in the title, abstract or list of keywords of all articles [18]. Moreover, some of the included papers did not have abstracts and or keywords [13], making it difficult to identify them through the search strategy.

In conclusion, cascade testing in the child-to-parent direction has been reported in a variety of diseases. This study examined 20 primary studies describing the uptake, yield and consequences of cascade testing triggered by genetic testing in a child. While most studies discussed the uptake and yield of testing, few addressed the costs and health system implications of cascade testing, so these areas remain poorly understood. Cascade effects are not currently considered in HTA and understanding the scope of cascade health service use will aid in the design and conduct of economic evaluations of emerging genetic testing technologies to more accurately assess their costs and benefits.

## Supplementary information

Table S1. Scoping literature review search strategy for Medline.

Table S2. Scoping literature review search strategy for Embase.

Supplementary File A. Critical appraisal methods and results.

Table S3. Critical appraisal of economic evaluations.

Table S4. Critical appraisal of cohort studies.

Video summary

## Data Availability

As a literature review, all data generated or analysed during this study are included in this published article (and its Supplementary information files).
