# Supplementary material for: Cascade health service use in family members following genetic testing in children: a scoping literature review
Source: Eur J Hum Genet. 2021 Aug 26;29(11):1601–10. doi: 10.1038/s41431-021-00952-4 (PMC8560854; doi:10.1038/s41431-021-00952-4)
Supplement: Supplementary file 2 — Table S2. Scoping literature review search strategy for Embase. [file 41431_2021_952_MOESM2_ESM.docx]

**Table S2.** Scoping literature review search strategy for Embase.

| **#** | **SEARCH TERMS** | **RECORDS RETRIEVED** |
| --- | --- | --- |
| 1. | exp genetic screening/ | 85,207 |
| 2. | exp genotyping technique/ | 8,667 |
| 3. | exp genotype/ | 443,014 |
| 4. | exp molecular diagnosis/ | 18,580 |
| 5. | exp molecular diagnostics/ | 7,132 |
| 6. | exp high throughput sequencing/ | 28,898 |
| 7. | exp sequence analysis/ | 236,304 |
| 8. | 1 or 2 or 3 or 4 or 5 or 6 or 7 | 768,725 |
| 9. | exp chromosome disorder/ | 66,157 |
| 10. | "genetic and familial disorders"/ | 68 |
| 11. | exp genetic predisposition/ | 157,593 |
| 12. | 9 or 10 or 11 | 223,040 |
| 13. | 8 and 12 | 58,561 |
| 14. | genetic screening.mp. [mp=title, abstract, heading word, drug trade name, original title, device manufacturer, drug manufacturer, device trade name, keyword, floating subheading word, candidate term word] | 86,214 |
| 15. | genotyping technique.mp. [mp=title, abstract, heading word, drug trade name, original title, device manufacturer, drug manufacturer, device trade name, keyword, floating subheading word, candidate term word] | 8,816 |
| 16. | genotype.mp. [mp=title, abstract, heading word, drug trade name, original title, device manufacturer, drug manufacturer, device trade name, keyword, floating subheading word, candidate term word] | 462,838 |
| 17. | molecular diagnosis.mp. [mp=title, abstract, heading word, drug trade name, original title, device manufacturer, drug manufacturer, device trade name, keyword, floating subheading word, candidate term word] | 25,224 |
| 18. | molecular diagnostics.mp. [mp=title, abstract, heading word, drug trade name, original title, device manufacturer, drug manufacturer, device trade name, keyword, floating subheading word, candidate term word] | 8,640 |
| 19. | high throughput sequencing.mp. [mp=title, abstract, heading word, drug trade name, original title, device manufacturer, drug manufacturer, device trade name, keyword, floating subheading word, candidate term word] | 33,832 |
| 20. | sequence analysis.mp. [mp=title, abstract, heading word, drug trade name, original title, device manufacturer, drug manufacturer, device trade name, keyword, floating subheading word, candidate term word] | 197,762 |
| 21. | next generation sequencing.mp. [mp=title, abstract, heading word, drug trade name, original title, device manufacturer, drug manufacturer, device trade name, keyword, floating subheading word, candidate term word] | 66,788 |
| 22. | Sanger sequencing.mp. [mp=title, abstract, heading word, drug trade name, original title, device manufacturer, drug manufacturer, device trade name, keyword, floating subheading word, candidate term word] | 26,076 |
| 23. | single gene test.mp. [mp=title, abstract, heading word, drug trade name, original title, device manufacturer, drug manufacturer, device trade name, keyword, floating subheading word, candidate term word] | 19 |
| 24. | gene panel.mp. [mp=title, abstract, heading word, drug trade name, original title, device manufacturer, drug manufacturer, device trade name, keyword, floating subheading word, candidate term word] | 4,736 |
| 25. | chromosome microarray.mp. [mp=title, abstract, heading word, drug trade name, original title, device manufacturer, drug manufacturer, device trade name, keyword, floating subheading word, candidate term word] | 432 |
| 26. | gene sequencing.mp. [mp=title, abstract, heading word, drug trade name, original title, device manufacturer, drug manufacturer, device trade name, keyword, floating subheading word, candidate term word] | 13,570 |
| 27. | genetic testing.mp. [mp=title, abstract, heading word, drug trade name, original title, device manufacturer, drug manufacturer, device trade name, keyword, floating subheading word, candidate term word] | 34,824 |
| 28. | 14 or 15 or 16 or 17 or 18 or 19 or 20 or 21 or 22 or 23 or 24 or 25 or 26 or 27 | 848,027 |
| 29. | chromosome disorder.mp. [mp=title, abstract, heading word, drug trade name, original title, device manufacturer, drug manufacturer, device trade name, keyword, floating subheading word, candidate term word] | 9,402 |
| 30. | (genetic and familial disorders).mp. [mp=title, abstract, heading word, drug trade name, original title, device manufacturer, drug manufacturer, device trade name, keyword, floating subheading word, candidate term word] | 162 |
| 31. | genetic predisposition.mp. [mp=title, abstract, heading word, drug trade name, original title, device manufacturer, drug manufacturer, device trade name, keyword, floating subheading word, candidate term word] | 65,927 |
| 32. | genetic disease.mp. [mp=title, abstract, heading word, drug trade name, original title, device manufacturer, drug manufacturer, device trade name, keyword, floating subheading word, candidate term word] | 10,457 |
| 33. | genetic anomaly.mp. [mp=title, abstract, heading word, drug trade name, original title, device manufacturer, drug manufacturer, device trade name, keyword, floating subheading word, candidate term word] | 348 |
| 34. | genetic condition.mp. [mp=title, abstract, heading word, drug trade name, original title, device manufacturer, drug manufacturer, device trade name, keyword, floating subheading word, candidate term word] | 2,081 |
| 35. | chromosome anomaly.mp. [mp=title, abstract, heading word, drug trade name, original title, device manufacturer, drug manufacturer, device trade name, keyword, floating subheading word, candidate term word] | 506 |
| 36. | chromosomal anomaly.mp. [mp=title, abstract, heading word, drug trade name, original title, device manufacturer, drug manufacturer, device trade name, keyword, floating subheading word, candidate term word] | 1,010 |
| 37. | inherited disease.mp. [mp=title, abstract, heading word, drug trade name, original title, device manufacturer, drug manufacturer, device trade name, keyword, floating subheading word, candidate term word] | 4,011 |
| 38. | inherited condition.mp. [mp=title, abstract, heading word, drug trade name, original title, device manufacturer, drug manufacturer, device trade name, keyword, floating subheading word, candidate term word] | 851 |
| 39. | hereditary disease.mp. [mp=title, abstract, heading word, drug trade name, original title, device manufacturer, drug manufacturer, device trade name, keyword, floating subheading word, candidate term word] | 3,416 |
| 40. | hereditary condition.mp. [mp=title, abstract, heading word, drug trade name, original title, device manufacturer, drug manufacturer, device trade name, keyword, floating subheading word, candidate term word] | 541 |
| 41. | genetic abnormality.mp. [mp=title, abstract, heading word, drug trade name, original title, device manufacturer, drug manufacturer, device trade name, keyword, floating subheading word, candidate term word] | 2,349 |
| 42. | chromosome abnormality.mp. [mp=title, abstract, heading word, drug trade name, original title, device manufacturer, drug manufacturer, device trade name, keyword, floating subheading word, candidate term word] | 2,290 |
| 43. | chromosomal abnormality.mp. [mp=title, abstract, heading word, drug trade name, original title, device manufacturer, drug manufacturer, device trade name, keyword, floating subheading word, candidate term word] | 4,680 |
| 44. | 29 or 30 or 31 or 32 or 33 or 34 or 35 or 36 or 37 or 38 or 39 or 40 or 41 or 42 or 43 | 106,367 |
| 45. | 28 and 44 | 25,287 |
| 46. | 13 or 45 | 67,191 |
| 47. | juvenile/ or exp adolescent/ or exp child/ or exp postnatal development/ or (pediatric* or paediatric* or child* or newborn* or congenital* or infan* or baby or babies or neonat* or pre term or preterm* or premature birth or NICU or preschool* or pre school* or kindergarten* or elementary school* or nursery school* or schoolchild* or toddler* or boy or boys or girl* or middle school* or pubescen* or juvenile* or teen* or youth* or high school* or adolesc* or prepubesc* or pre pubesc*).mp. or (child* or adolesc* or pediat* or paediat*).jn. | 5,256,135 |
| 48. | proband.mp. [mp=title, abstract, heading word, drug trade name, original title, device manufacturer, drug manufacturer, device trade name, keyword, floating subheading word, candidate term word] | 15,018 |
| 49. | index patient.mp. [mp=title, abstract, heading word, drug trade name, original title, device manufacturer, drug manufacturer, device trade name, keyword, floating subheading word, candidate term word] | 3,131 |
| 50. | index case.mp. [mp=title, abstract, heading word, drug trade name, original title, device manufacturer, drug manufacturer, device trade name, keyword, floating subheading word, candidate term word] | 5,895 |
| 51. | 48 or 49 or 50 | 23,641 |
| 52. | 47 and 51 | 11,850 |
| 53. | 46 and 52 | 468 |
| 54. | exp heterozygote detection/ | 6,563 |
| 55. | exp family/ | 552,841 |
| 56. | 54 and 55 | 477 |
| 57. | heterozygote detection.mp. [mp=title, abstract, heading word, drug trade name, original title, device manufacturer, drug manufacturer, device trade name, keyword, floating subheading word, candidate term word] | 6,676 |
| 58. | predictive testing.mp. [mp=title, abstract, heading word, drug trade name, original title, device manufacturer, drug manufacturer, device trade name, keyword, floating subheading word, candidate term word] | 1,417 |
| 59. | preventive testing.mp. [mp=title, abstract, heading word, drug trade name, original title, device manufacturer, drug manufacturer, device trade name, keyword, floating subheading word, candidate term word] | 30 |
| 60. | carrier testing.mp. [mp=title, abstract, heading word, drug trade name, original title, device manufacturer, drug manufacturer, device trade name, keyword, floating subheading word, candidate term word] | 769 |
| 61. | predictive screening.mp. [mp=title, abstract, heading word, drug trade name, original title, device manufacturer, drug manufacturer, device trade name, keyword, floating subheading word, candidate term word] | 153 |
| 62. | preventive screening.mp. [mp=title, abstract, heading word, drug trade name, original title, device manufacturer, drug manufacturer, device trade name, keyword, floating subheading word, candidate term word] | 577 |
| 63. | carrier screening.mp. [mp=title, abstract, heading word, drug trade name, original title, device manufacturer, drug manufacturer, device trade name, keyword, floating subheading word, candidate term word] | 1,547 |
| 64. | cascade testing.mp. [mp=title, abstract, heading word, drug trade name, original title, device manufacturer, drug manufacturer, device trade name, keyword, floating subheading word, candidate term word] | 361 |
| 65. | cascade screening.mp. [mp=title, abstract, heading word, drug trade name, original title, device manufacturer, drug manufacturer, device trade name, keyword, floating subheading word, candidate term word] | 621 |
| 66. | reverse cascade testing.mp. [mp=title, abstract, heading word, drug trade name, original title, device manufacturer, drug manufacturer, device trade name, keyword, floating subheading word, candidate term word] | 3 |
| 67. | reverse cascade screening.mp. [mp=title, abstract, heading word, drug trade name, original title, device manufacturer, drug manufacturer, device trade name, keyword, floating subheading word, candidate term word] | 9 |
| 68. | familial testing.mp. [mp=title, abstract, heading word, drug trade name, original title, device manufacturer, drug manufacturer, device trade name, keyword, floating subheading word, candidate term word] | 32 |
| 69. | familial screening.mp. [mp=title, abstract, heading word, drug trade name, original title, device manufacturer, drug manufacturer, device trade name, keyword, floating subheading word, candidate term word] | 328 |
| 70. | familial mutation analysis.mp. [mp=title, abstract, heading word, drug trade name, original title, device manufacturer, drug manufacturer, device trade name, keyword, floating subheading word, candidate term word] | 3 |
| 71. | surveillance strategy.mp. [mp=title, abstract, heading word, drug trade name, original title, device manufacturer, drug manufacturer, device trade name, keyword, floating subheading word, candidate term word] | 1,017 |
| 72. | surveillance program.mp. [mp=title, abstract, heading word, drug trade name, original title, device manufacturer, drug manufacturer, device trade name, keyword, floating subheading word, candidate term word] | 32,434 |
| 73. | 57 or 58 or 59 or 60 or 61 or 62 or 63 or 64 or 65 or 66 or 67 or 68 or 69 or 70 or 71 or 72 | 45,102 |
| 74. | family.mp. [mp=title, abstract, heading word, drug trade name, original title, device manufacturer, drug manufacturer, device trade name, keyword, floating subheading word, candidate term word] | 1,197,221 |
| 75. | first-degree relatives.mp. [mp=title, abstract, heading word, drug trade name, original title, device manufacturer, drug manufacturer, device trade name, keyword, floating subheading word, candidate term word] | 14,006 |
| 76. | parents.mp. [mp=title, abstract, heading word, drug trade name, original title, device manufacturer, drug manufacturer, device trade name, keyword, floating subheading word, candidate term word] | 235,532 |
| 77. | siblings.mp. [mp=title, abstract, heading word, drug trade name, original title, device manufacturer, drug manufacturer, device trade name, keyword, floating subheading word, candidate term word] | 50,181 |
| 78. | grandparents.mp. [mp=title, abstract, heading word, drug trade name, original title, device manufacturer, drug manufacturer, device trade name, keyword, floating subheading word, candidate term word] | 3,321 |
| 79. | relative.mp. [mp=title, abstract, heading word, drug trade name, original title, device manufacturer, drug manufacturer, device trade name, keyword, floating subheading word, candidate term word] | 1,072,430 |
| 80. | father.mp. [mp=title, abstract, heading word, drug trade name, original title, device manufacturer, drug manufacturer, device trade name, keyword, floating subheading word, candidate term word] | 50,952 |
| 81. | mother.mp. [mp=title, abstract, heading word, drug trade name, original title, device manufacturer, drug manufacturer, device trade name, keyword, floating subheading word, candidate term word] | 235,020 |
| 82. | brother.mp. [mp=title, abstract, heading word, drug trade name, original title, device manufacturer, drug manufacturer, device trade name, keyword, floating subheading word, candidate term word] | 14,727 |
| 83. | sister.mp. [mp=title, abstract, heading word, drug trade name, original title, device manufacturer, drug manufacturer, device trade name, keyword, floating subheading word, candidate term word] | 41,891 |
| 84. | 74 or 75 or 76 or 77 or 78 or 79 or 80 or 81 or 82 or 83 | 2,627,671 |
| 85. | at-risk.mp. [mp=title, abstract, heading word, drug trade name, original title, device manufacturer, drug manufacturer, device trade name, keyword, floating subheading word, candidate term word] | 251,029 |
| 86. | 84 and 85 | 40,384 |
| 87. | 73 and 86 | 746 |
| 88. | 56 or 87 | 1,185 |
| 89. | 46 and 53 and 88 | 5 |
| 90. | limit 89 to (english language and yr="2000 -Current") | 5 |

Search line 47 is a filter to retrieve paediatrics articles in Ovid Embase developed at the University of Alberta [1].

1. Desmeules R. Filter to retrieve pediatrics articles in OVID Embase. University of Alberta, 2018. [Internet]. Available from https://guides.library.ualberta.ca/c.php?g=342568&p=5096194.
