# Supplementary material for: Cascade health service use in family members following genetic testing in children: a scoping literature review
Source: Eur J Hum Genet. 2021 Aug 26;29(11):1601–10. doi: 10.1038/s41431-021-00952-4 (PMC8560854; doi:10.1038/s41431-021-00952-4)
Supplement: Supplementary file 3 — Supplementary File A. Critical appraisal methods and results. [file 41431_2021_952_MOESM3_ESM.docx]

**Supplementary File A: Critical Appraisal**

**MATERIALS AND METHODS**

Included papers were critically appraised using the Scottish Intercollegiate Guidelines Network (SIGN) critical appraisal checklist appropriate to the study design. The quality of included case reports and case series was not appraised, in accordance with the SIGN Algorithm for Classifying Study Design for Questions of Effectiveness, with the understanding that these types of studies are of lower quality than empiric observational studies. The overall quality of each study was reported as *high*, *acceptable*, or *low quality*. The goal of this review was to report on all available literature, so no study was excluded on the basis of quality.

**RESULTS**

Studies were categorized as *high quality*, *acceptable*, or *low quality* (**Supplementary Tables S3** and **S4**). The majority of appraised studies were acceptable, with one [1] considered low quality. Most cohort studies were non-comparative, and two were retrospective [2, 3]. Consequently, many of the criteria on the checklist related to prospective comparative studies were not applicable. None of the appraised publications could be classified as *high quality* as “retrospective studies or single cohort studies are generally regarded as weaker design and should not receive a rating higher than [acceptable]” [4]. In general, studies stated their objective well, described their methodology in sufficient detail, and reported primary and secondary findings. Several studies lacked information about their participant selection process and did not provide adequate demographic information [1, 5-9]. One [1] was rated as low quality because the methodology was unclear and lacking in detail.

Although case reports [10-14] and case series [15-17] were not subjected to critical appraisal, it was recognized that they were inherently of poorer quality than cohort studies. In general case reports and case series suffered from a number of design flaws, including lack of a control group and inherent selection bias [18]. A common weakness was a failure to discuss implications for care [11-13]. None of the included case series adequately described their methodology for patient identification or criteria for inclusion [15-17].

**REFERENCES**

1. Gorakshakar AC, Colah RB. Cascade screening for beta-thalassemia: A practical approach for identifying and counseling carriers in India. *Indian J Community Med*. 2009;**34**(4):354-356.

2. Knight LM, Miller E, Kovach J, Arscott P, von Alvensleben JC, Bradley D, et al. Genetic testing and cascade screening in pediatric long QT syndrome and hypertrophic cardiomyopathy. *Heart Rhythm*. 2020;**17**(1):106-112.

3. Miller EM, Wang Y, Ware SM. Uptake of cardiac screening and genetic testing among hypertrophic and dilated cardiomyopathy families. *J Genet Couns*. 2013;**22**(2):258-267.

4. Scottish Intercollegiate Guidelines Network (SIGN). Critical appraisal notes and checklists [Internet]. Available from: https://www.sign.ac.uk/checklists-and-notes.

5. Cadet E, Capron D, Gallet M, Omanga-Leke ML, Boutignon H, Julier C, et al. Reverse cascade screening of newborns for hereditary haemochromatosis: a model for other late onset diseases? *J Med Genet*. 2005;**42**(5):390-395.

6. McClaren BJ, Metcalfe SA, Aitken M, Massie RJ, Ukoumunne OC, Amor DJ. Uptake of carrier testing in families after cystic fibrosis diagnosis through newborn screening. *Eur J Hum Genet*. 2010;**18**(10):1084-1089.

7. Smith M, Calabro V, Chong B, Gardiner N, Cowie S, du Sart D. Population screening and cascade testing for carriers of SMA. *Eur J Hum Genet*. 2007;**15**(7):759-766.

8. Stark Z, Schofield D, Martyn M, Rynehart L, Shrestha R, Alam K, et al. Does genomic sequencing early in the diagnostic trajectory make a difference? A follow-up study of clinical outcomes and cost-effectiveness. *Genet Med*. 2019;**21**(1):173-180.

9. Wu X, Pang J, Wang X, Peng J, Chen Y, Wang S, et al. Reverse cascade screening for familial hypercholesterolemia in high-risk Chinese families. *Clin Cardiol*. 2017;**40**(11):1169-1173.

10. Famula J, Basuta K, Gane LW, Hagerman RJ, Tassone F. Identification of a male with fragile X syndrome through newborn screening. *Intractable Rare Dis Res*. 2015;**4**(4):198-202.

11. Sorensen PL, Basuta K, Mendoza-Morales G, Gane LW, Schneider A, Hagerman R, et al. A fragile X sibship from a consanguineous family with a compound heterozygous female and partially methylated full mutation male. *Am J Med Genet A*. 2012;**158A**(5):1221-1224.

12. Tairaku S, Taniguchi-Ikeda M, Okazaki Y, Noguchi Y, Nakamachi Y, Mori T, et al. Prenatal genetic testing for familial severe congenital protein C deficiency. *Hum Genome Var*. 2015;**2**:15017.

13. Baig SM, Din MA, Hassan H, Azhar A, Baig JM, Aslam M, et al. Prevention of beta-thalassemia in a large Pakistani family through cascade testing. *Community Genet*. 2008;**11**(1):68-70.

14. Rudolph G, Meindl A, Bechmann M, Schworm HD, Achatz H, Boergen KP, et al. X-linked ocular albinism (Nettleship-Falls): a novel 29-bp deletion in exon 1. Carrier detection by ophthalmic examination and DNA analysis. *Graefes Arch Clin Exp Ophthalmol*. 2001;**239**(3):167-172.

15. Moriwaki S, Yamashita Y, Nakamura S, Fujita D, Kohyama J, Takigawa M, et al. Prenatal diagnosis of xeroderma pigmentosum group A in Japan. *J Dermatol*. 2012;**39**(6):516-519.

16. Sorensen PL, Gane LW, Yarborough M, Hagerman RJ, Tassone F. Newborn screening and cascade testing for FMR1 mutations. *Am J Med Genet A*. 2013;**161A**(1):59-69.

17. Truong TH, Kim NT, Nguyen MNT, Pang J, Hooper AJ, Watts GF, et al. Homozygous familial hypercholesterolaemia in Vietnam: case series, genetics and cascade testing of families. *Atherosclerosis*. 2018;**277**:392-398.

18. Sayre JW, Toklu HZ, Ye F, Mazza J, Yale S. Case reports, case series - from clinical practice to evidence-based medicine in graduate medical education. *Cureus*. 2017;**9**(8):e1546.
