# Supplementary material for: Cascade health service use in family members following genetic testing in children: a scoping literature review
Source: Eur J Hum Genet. 2021 Aug 26;29(11):1601–10. doi: 10.1038/s41431-021-00952-4 (PMC8560854; doi:10.1038/s41431-021-00952-4)
Supplement: Supplementary file 4 — Table S3. Critical appraisal of economic evaluations. [file 41431_2021_952_MOESM4_ESM.docx]

**Table S3.** Critical appraisal of economic evaluations.

|  | | **REFERENCE** |
| --- | --- | --- |
| **CRITERION** | | Stark *et al*., 2019 [19] |
| 1. | The study addresses an appropriate and clearly focused question. | Y |
| 2. | The economic importance of the question is clear. | Y |
| 3. | The choice of study design is justified. | N |
| 4. | All costs that are relevant from the viewpoint of the study are included and measured and valued appropriately. | N |
| 5. | The outcome measures used to answer the study question are relevant to that purpose and are measured and valued appropriately. | Y |
| 6. | If discounting future costs and outcomes is necessary, it has been performed correctly. | NA |
| 7. | Assumptions are made explicit and a sensitivity analysis was performed. | C |
| 8. | The decision rule is made explicit and comparisons are made on the basis of incremental costs and outcomes. | Y |
| 9. | The results provide information of relevance to policy makers. | Y |
| **OVERALL ASSESSMENT** | | **A** |

*Y: yes N: no C: cannot say A: acceptable NA: not applicable*
