# Supplementary material for: Cascade health service use in family members following genetic testing in children: a scoping literature review
Source: Eur J Hum Genet. 2021 Aug 26;29(11):1601–10. doi: 10.1038/s41431-021-00952-4 (PMC8560854; doi:10.1038/s41431-021-00952-4)
Supplement: Supplementary file 5 — Table S4. Critical appraisal of cohort studies. [file 41431_2021_952_MOESM5_ESM.docx]

**Table S4.** Critical appraisal of cohort studies.

|  | | **REFERENCE** | | | | | | | | | | |
| --- | --- | --- | --- | --- | --- | --- | --- | --- | --- | --- | --- | --- |
| **CRITERION** | | Knight *et al*., 2020 [16] | Wu *et al*., 2017 [15] | Wald *et al*., 2016 [20] | Alfares *et al*., 2015 [18] | McClaren *et al*., 2013 [17] | Miller *et al*., 2013 [24] | McClaren *et al*., 2010 [14] | Gorakshakar & Colah, 2009 [13] | Smith *et al*., 2007 [31] | Cadet *et al*., 2005 [28] | Leren *et al*., 2004 [25] |
| 1. | The study addresses an appropriate and clearly focused question. | Y | Y | Y | Y | Y | Y | Y | C | C | Y | C |
| 2. | The two groups being studied are selected from source populations that are comparable in all respects other than the factor under investigation. | NA | NA | NA | NA | NA | NA | NA | NA | C | NA | NA |
| 3. | The study indicates how many of the people asked to take part did so, in each of the groups being studied. | NA | NA | NA | NA | NA | NA | NA | NA | N | NA | NA |
| 4. | The likelihood that some eligible subjects might have the outcome at the time of enrolment is assessed and taken into account in the analysis. | NA | NA | NA | NA | NA | NA | NA | NA | NA | NA | NA |
| 5. | What percentage of individuals or clusters recruited into each arm of the study dropped out before the study was completed? | NA | NA | NA | NA | NA | NA | NA | NA | NA | NA | NA |
| 6. | Comparison is made between full participants and those lost to follow-up, by exposure status. | NA | NA | NA | NA | NA | NA | NA | NA | NA | NA | NA |
| 7. | The outcomes are clearly defined. | Y | Y | Y | Y | Y | Y | Y | Y | Y | Y | Y |
| 8. | The assessment of outcome is made blind to exposure status. If the study is retrospective, this may not be applicable. | NA | NA | NA | NA | NA | NA | NA | NA | NA | NA | NA |
| 9. | Where blinding was not possible, there is some recognition that knowledge of exposure status could have influenced the assessment of outcome. | NA | NA | NA | NA | NA | NA | NA | NA | NA | NA | NA |
| 10. | The method of assessment of exposure is reliable. | NA | NA | NA | NA | NA | NA | NA | NA | NA | NA | NA |
| 11. | Evidence from other sources is used to demonstrate that the method of outcome assessment is valid and reliable. | C | Y | C | Y | NA | N | N | N | Y | N | Y |
| 12. | Exposure level or prognostic factor is assessed more than once. | NA | NA | NA | NA | NA | NA | NA | NA | NA | NA | NA |
| 13. | The main potential confounders are identified and taken into account in the design and analysis. | NA | NA | N | NA | Y | Y | Y | NA | NA | NA | Y |
| 14. | Have confidence intervals been provided? | Y | Y | Y | Y | Y | Y | Y | Y | Y | N | Y |
| **OVERALL ASSESSMENT** | | **A** | **A** | **A** | **A** | **A** | **A** | **A** | **LQ** | **A** | **A** | **A** |

*Y: yes N: no C: cannot say HQ: high quality A: acceptable LQ: low quality NA: not applicable*
